# Stabilizing cathode structure *via* the binder material with high resilience for lithium–sulfur batteries†

**DOI:** 10.1039/c9ra08238g

**Published:** 2019-12-06

**Authors:** Fengquan Liu, Zhiyu Hu, Jinxin Xue, Hong Huo, Jianjun Zhou, Lin Li

**Affiliations:** Beijing Key Laboratory of Energy Conversion and Storage Materials, College of Chemistry, Beijing Normal University Beijing 100875 P. R. China pla_zjj@bnu.edu.cn lilinll@bnu.edu.cn

## Abstract

Lithium–sulfur (Li–S) batteries have been considered as one of the most promising next-generation energy storage systems with high-energy density. The huge volumetric change of sulfur (*ca.* 80% increase in volume) in the cathode during discharge is one of the factors affecting the battery performance, which can be remedied with a binder. Herein, a self-crosslinking polyacrylate latex (PAL) is synthesized and used as a binder for the sulfur cathode of a Li–S battery to keep the cathode structure stable. The synthesized PAL has nano-sized latex particles and a low glass transition temperature (*T*_g_), which will ensure a uniform dispersion and good adhesion in the cathode. This crosslinking structure can provide fine elasticity to recover from the deformation due to volumetric change. The stable cathode structure, stemming from the fine elasticity of the PAL binder, can facilitate ion migration and diffusion to decrease the polarization. Therefore, the Li–S batteries with the PAL binder can function well with excellent cycling stability and superior C-rate performance.

## Abstract 2

A self-crosslinking polyacrylate binder with fine elasticity stabilizing the sulfur cathode and endowing Li–S batteries with excellent performance.

## Graphical abstract

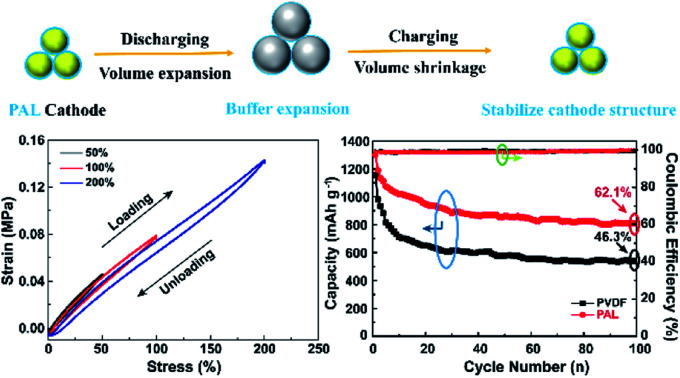

## Introduction

Currently, with the development of portable electronic devices, electric vehicles, and smart grids, great attention has been paid to lithium ion secondary batteries with high-energy density and a long lifespan.^1–6^ The high theoretical capacity of lithium–sulfur (Li–S) batteries will meet the increasing demand for higher energy storage devices.^7–9^ There is no doubt that sulfur is a promising candidate for a cathode material because of the many advantages such as its natural abundance, low cost, low toxicity and environmental friendliness.^10–12^ However, the wide application of Li–S batteries still suffers from some defects, which lead to poor cycling stability and rate capacity.^13,14^ The defects mentioned above include: electrical insulating nature of elemental sulfur and its insoluble discharge products Li_2_S_2_ and Li_2_S^15,16^ as well as the growth of Li dendrites^17–19^ and the “shuttle effect” originating from the dissolution of the intermediate polysulfides in organic electrolytes.^20–23^ To surmount the aforementioned defects, numerous efforts have been made to improve the performance of Li–S battery. Various kinds of sulfur contained cathode material have been designed, for example: sulfur attached on the surface of conductive carbon materials,^24–26^ sulfur embedded into the mesoporous carbon/spherical carbon,^25,27,28^ polysulfide fixed sulfur with chemical bonds,^29^ “sandwich” type cathode material,^30,31^ composite cathode by reacting sulfur with biomass carbon^32^ and construction of fast ions transport pathways within the inner of electrodes.^33–37^ Most studies mainly focus on the microstructure of carbon and sulfur, whereas preservation of the cathode structure consisting of microstructure is crucial to the performance of Li–S batteries. Due to the conversion character of sulfur and its huge volumetric change during cycling, the cathode structure will also undergo great changes. The cathode is prepared by adhering the active material, conductive carbon and collector together with a binder. Although the content of the binder is low, it plays an important role in stabilizing the cathode structure.

Polyvinylidene fluoride (PVDF) is the most popular binder for cathodes in traditional lithium ion batteries (LIBs) due to its great advantages, including superior electrochemical stability, high adhesion to electrode materials and current collectors. However, many problems also exist and need to be solved for PVDF in Li–S battery due to its poor interaction with polysulfides, inferior buffer capacity to the volume expansion for cathode, toxic and relative expensive of solvent *etc.*^38^ Recently, Yan *et al.* synthesized a series of novel binder with amino functional group, exhibiting strong absorption ability to lithium polysufides, which improved cycling performance of the Li–S batteries obviously.^39,40^ Moreover, lots of efforts have been made to the pursuit of environment-friendly, cost-effective, aqueous-based binder system for Li–S battery. Studies have confirmed that many water-soluble polymers used as binders can improve the electrochemical performance.^41–48^ For example, Fu *et al.* designed and developed a multiple binder, which incorporate soy protein (SP) with PAA. The functional groups of SP have strong capability to adsorb polysulfides, enhancing the Li–S battery electrochemical performances. A binder with high electronic conductive and strong adsorption was also fabricated *via* combining reduced graphene oxide with polyacrylic acid (GO-PAA), reducing the local charge-transfer resistance and the global electronic resistance.^47^ Furthermore, Zhou *et al.*^49^ reported an aqueous inorganic polymer, ammonium polyphosphate (APP), which can not only promote ion transfer and accelerate the reaction kinetics for the sulfur cathode, but also prominently reduce the flammability of sulfur cathode to improve safety. The radical polymer poly(2,2,6,6-tetramethyl-1-piperidinyloxy-4-yl methacrylate) was studied as a stable binder, that can provide additional redox sites and promote the reaction kinetics of sulfur cathode.^50^ Wang *et al.*^51^ developed a modified β-cyclodextrin (β-CD) as a binder with strong bonding capability and superior mechanical properties, endowing the Li–S batteries with excellent electrochemical stability.

Although great progress has been made, a binder with improving performance is urgently needed. The sulfur in the cathode is reduced to Li_2_S or Li_2_S_2_ during discharge, resulting in large volume change during the conversion reaction (*ca.* 80% increase in volume).^52,53^ More than 20% expansion and shrinkage of cathode thickness has been examined by using direct measurement with a micrometer instrument.^54^ Similar thickness change is proved by the *in situ* X-ray tomography in the first several cycles. In a large format Li–S pouch cell with multiple layers of electrodes stacked together, the cell thickness variation is found to change several hundred micrometers at the working voltage.^55^ With the result of the large volumetric expanding or shrinking in the cathode, the binder for Li–S batteries should possess not only strong adhesion but also high resilience. The strong adhesion can ensure that intimate contact is made amidst the active sulfur, conductive carbon and current collector. The high resilience can endure large volume change periodically during cycling to prevent the cathode material from falling off and maintain its structure stability.

Herein, a self-crosslinking polyacrylate latex (PAL) is synthesized and used as a novel binder for sulfur cathode *via* emulsion polymerization. The obtained binder has a low glass transition temperature that provides a strong adhesion. The crosslinking structure with fine elasticity links the cathode material and the current collector together, keeping the cathode structure stable. This work offers a feasible and effective strategy for designing and developing binder toward the Li–S batteries.

## Experimental section

### Synthesis of PAL

The PAL was synthesized with a two-stage semi-continuous emulsion polymerization.^56^ The detail processes were described as follows. At the first stage, in a 100 mL three-necked glass flask equipped with reflux condenser and dropping funnels, 3.20 g butyl acrylate (BA) monomer was pre-emulsified by emulsifier aqueous solution (0.48 g nonylphenolethoxylates (OP-10) and 0.48 g sodium dodecyl sulfate (SDS) dissolved in 41.00 g H_2_O) under vigorously stirring with Teflon mechanical stirrer at a speed of about 600 rpm for 30 min to form the pre-emulsion A. In another glass flask, 12.96 g 2-ethylhexyl acrylate (EHA), 10.00 g BA, 4.32 g methyl methacrylate (MMA), 0.72 g acrylic acid (AA) and 0.72 g diacetone acrylamide (DAAM) were pre-emulsified by emulsifier aqueous solution (0.16 g OP-10 and 0.16 g SDS in 22.4 g H_2_O) under energetically stirring with Teflon mechanical stirrer at a speed of about 600 rpm for at least 30 min to form the pre-emulsion B. At the second stage, the pre-emulsion A was heated to 82 °C in a water bath under a stirring speed of about 280 rpm, and then the ammonium persulfate (APS) aqueous solution (0.42 g APS dissolved in 1.5 g H_2_O) was added to initiate polymerizing. The solution became blue after reacting for about 3 min. 30 min later, the pre-emulsion B was added dropwise. When the feeding was completed, the polymerization was allowed to react for another 1 h. The latex was cooled to 45 °C, and the pH value was adjusted to about 8 by NH_3_·H_2_O. Finally, 0.72 g adipicdihydrazide (ADH) was added as crosslinking agent to obtain the crosslinking PAL with solid content of about 40 wt%. As NH_3_·H_2_O evaporated at the drying process, the neutral carboxyl group in AA unit of PAL would recover to acid status with hydrogen ion, which led to the crosslinking reaction of ADH and DAAM in acid condition.

### Electrode preparation

Pure sulfur (analytical reagent, Sigma-Aldrich) was mixed with Super P (SP) according to the quality ratio of 7.5 : 2.5. The mixtures were then sealed in a glass container and heated at 155 °C for 20 h to make sulfur and SP disperse fully. The SP/S composite which contains 72.9 wt% of sulfur was acquired after being natural cooled to room temperature (Fig. S1†).

The cathode slurry was prepared by mixing 80 wt% SP/S, 10 wt% Super P and 10 wt% PAL binder in ethanol/water (v/v = 1/4). The slurry of the cathode was coated onto a sheet of aluminum foil. The electrode was dried under vacuum at 60 °C for 24 h before using. The sulfur content in the cathode was 52.1 wt% after vacuum drying (Fig. S2†). For comparison, cathode electrode with PVDF as a binder was also prepared by the similar routes. The same composition ratio was used except NMP was used as solvent for PVDF. The sulfur loading of the as-prepared cathode was about 1.5 mg cm^−2^.

### Material characterization

PAL sample for morphological study was prepared by diluting with distilled water, and then dropped on carbon-coated copper grid and dried under vacuum oven at 40 °C for 10 h. The morphology was observed with transmission electron microscopy (TEM) (JEOL JEM-2100). PAL film was obtained by direct drying to investigate the glass transition temperature (*T*_g_) and its chemical structure. *T*_g_ was measured with differential scanning calorimeter (DSC) (DSC 8000, PerkinElmer Corp.) at a heating rate of 10 °C min. Chemical structure of PAL was investigated with Fourier transmission infrared spectroscopy (FTIR) (Nicolet Avatar 360). The morphologies of cathodes were observed by a scanning electron microscope (SEM) (Hitachi SU8010). Thermogravimetric analysis of SP/S was recorded to acquire the sulfur content in the composite materials with a heating rate of 10 K min^−1^ from 25 to 850 °C under N_2_ atmosphere. Tensile mechanical properties of the PAL and PVDF films were determined using an electronic universal testing machine (Instron 3366, Instron Corporation, USA) with a 100 N load cell at the cross-head speed of 100 mm min^−1^.

### Cell assembly and characterization

CR2032-type coin cell was fabricated in an argon-filled glove box (H_2_O < 0.1 ppm, O_2_ < 0.1 ppm). The electrolyte was 1 M bis(trifluoromethane) sulfonamide lithium (LiTFSI, Sigma Aldrich) in a mixed solvent of 1,2-dimethoxyethane (DME, Sigma Aldrich) and 1,3-dioxolane (DOL, Sigma Aldrich) (1/1, v/v) with 1 wt% lithium nitrate (LiNO_3_, Sigma Aldrich) as additive. The ratio of electrolyte to sulfur is about 16. Lithium foil was used as counter electrode and a 20 μm polypropylene separator (CangzhouMingzhu Plastic Co, Ltd, China) was used. Electrochemical impedance spectroscopy (EIS) tests were measured at open-circuit potential (OCP) in the frequency range of 100 kHz–0.1 Hz. Cyclic voltammetry (CV) tests were performed at different scan rates (0.1, 0.2, 0.5, 1.0 and 2.0 mV s^−1^). Galvanostatic charge/discharge tests were completed in the potential range between 1.8 V and 2.8 V with a Land CT2001A battery-testing instrument.

## Results

**Fig. 1:**
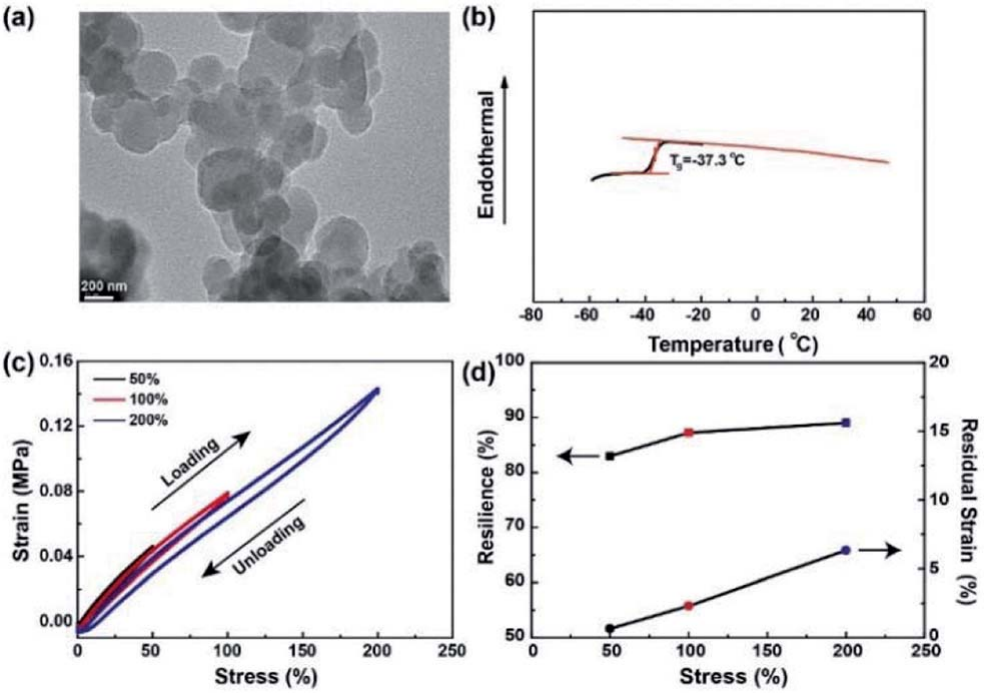
(a) The TEM morphology of PAL particles. (b) The DSC heating curve of PAL film. (c) Cyclic loading–unloading curves of PAL film. (d) The resilience (square) and residual strains (circle) of PAL film to different tensile strains.

PAL is a low viscosity latex (∼58 mPa s at 60 rpm) with fine particles dispersed in water. The morphology of PAL particles is characterized by TEM, with the spherical latex particles having a size of around 500 nm (Fig. 1a). Low viscosity and nano-sized PAL particles prove to be helpful for a uniform dispersion in cathode materials. *T*_g_ of the PAL film is about −37 °C as shown in Fig. 1b, and the low *T*_g_ can ensure good adhesion to the cathode materials, conductive agent and Al current collector. FTIR is used to characterize the chemical constitution of the PAL film (Fig. S3†). The peak of 1721 cm^−1^ can be assigned to C=O stretching vibrations of acylamino.^57^ The characteristic adsorption peaks of approximately 3346 and 3269 cm^−1^ refer to the stretching vibration of –NH_2_. Moreover, the new peak at 1671 cm^−1^ can be attributed to the –C=N– after the film is dried, indicating the reaction of ketocarbonyl with hydrazine and the formation of cross-linked polymer.^48^

As we all know that the maximum volume change for sulfur cathode during cycling is about 80%.^42,43^ As stated, it is imperative to evaluate the capacity of binders to volume changes during charging/discharging by cyclic tensile tests. The loading and unloading cycles are applied for five times in immediate succession with the same specimen to increasing levels of maximum tensile strains. Very small hysteresis for PAL film are noticed between the loading and unloading curves as shown in Fig. 1c. In addition, at the same strain, the stress of the loading process is slightly higher than that of the previous unloading process. The resilience is calculated from the ratio of the area of the unloading curve to that of the loading curve and the residual strain is summarized in Fig. 1d. The PAL film exhibits very high resilience (around 89%) and very low residual strain (only 6.3% at 200% strain), suggesting that nearly no permanent damage has been done to the film in the cyclic tensile tests. In contrast, the cyclic loading and unloading curves of PVDF film show clear hysteresis phenomenon and very low resilience (∼2%) when the maximum tensile strain is only 50% (Fig. S4†). As can be seen from the above results, the PAL binder has a higher elasticity to tolerate the volume changes of the sulfur cathode than that of the PVDF during charging/discharging. The PAL binder can be swollen by the electrolyte with the size increase by 40% (Fig. S5†). The presence of liquid electrolyte will decrease the tensile strength, but the resilience stemming from crosslinking might be kept. Besides, CV scan has showed that the PAL is stable during cycling (Fig. S6†). Due to the excellent elasticity and electrochemical stability of the PAL, it is used as a binder for Li–S batteries to study its effect on cathode structure compared with the PVDF binder.

**Fig. 2:**
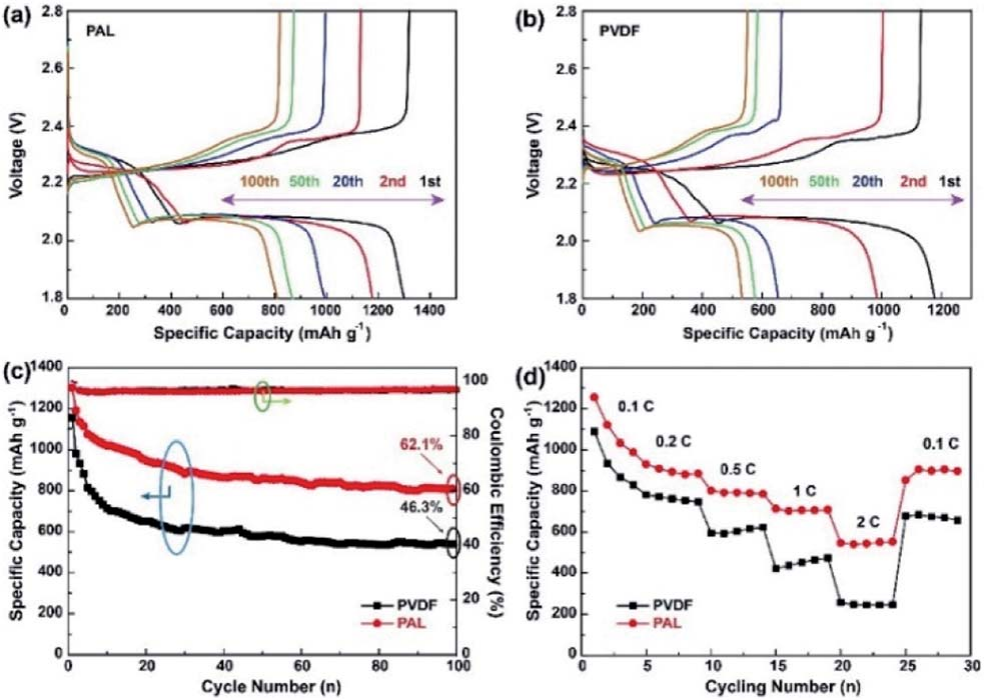
The charge/discharge profiles of (a) PAL and (b) PVDF batteries at the voltage of 1.8–2.8 V. (c) cycling and (d) C-rate performances of the PAL and PVDF batteries.

Fig. 2a and b display the galvanostatic charge/discharge voltage profiles of Li–S batteries with PAL and PVDF binders at a C-rate of 0.1. For simplicity, Li–S batteries with PAL and PVDF binders are designated as PAL and PVDF batteries. Two typical discharge plateaus exist, which could be attributed to the two step reactions of sulfur with Li during the discharge process.^14,58,59^ The lower fading rate of the PAL battery can be identified from the capacity difference between various cycles. The capacity loss is about 190 mA h g^−1^ during the 19 cycles between 2^nd^ and 20^th^ cycle in the PAL battery, much smaller than that in the PVDF battery (∼335 mA h g^−1^). The cycling stability and Coulombic efficiency (CE) are shown in Fig. 2c. The CE is about 99% for both kinds of batteries due to the addition of LiNO_3_. In the 1^st^ and 100^th^ cycles, the PAL battery delivers higher discharge capacity. Notably, the capacity retention ratio of PAL battery (62.1%) is obviously higher than that of PVDF battery (46.3%) after 100 cycles. The rate capacity performance is evaluated by increasing the C-rate from 0.1 to 2C (Fig. 2d). During the first several cycles, the discharge capacities fade gradually at 0.1C. The capacities further decrease as the C-rate increases. The discharge capacities are about 552.0 and 245.1 mA h g^−1^, respectively, corresponding to the PAL and PVDF batteries at 2C. The obvious larger discharge capacity is found in the PAL battery. As the C-rate returned to 0.1C, the capacities recover to a higher discharge capacity of 897.1 mA h g^−1^ from the PAL battery verses 657.3 mA h g^−1^ which was identified in the PVDF battery. Whether at a high or low C-rate, the PAL battery possesses better C-rate performance, indicating a distinct advantage of the PAL over PVDF binder. Moreover, the PAL battery presents better cycle stability than other binders reported in related literatures (Table S1†).

## Discussion

**Fig. 3:**
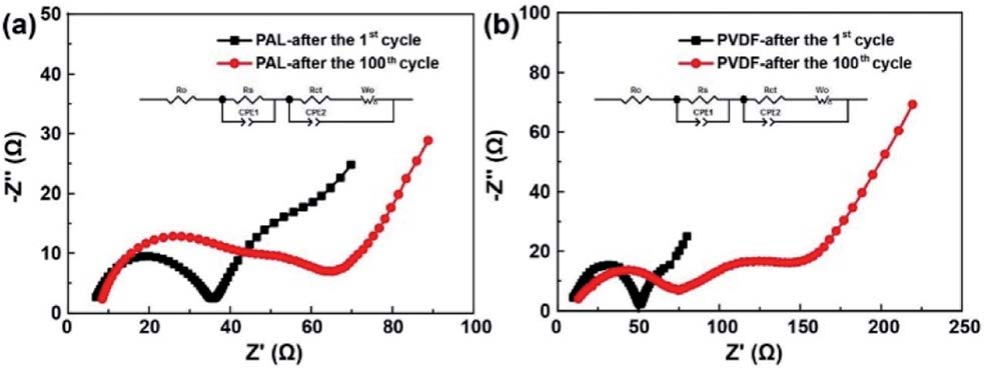
The EIS spectra of (a) the PAL and (b) PVDF batteries after the 1^st^ cycle and 100^th^ cycle.

EIS is conducted to explore what roles the PAL binder has played in improving the electrochemical performances. The Nyquist plots are shown in Fig. 3. A semicircle at high frequency, a semicircle at medium frequency and an inclined line in the low frequency region are detected.^60–62^ The high-frequency intercepting the real axis represents ohmic resistance (*R*_0_). The semicircle at high frequency corresponds to the resistance (*R*_s_) at the solid electrode interface. The semicircle at medium frequency is assigned to the charge-transfer resistance (*R*_ct_) occurring at the electrode. After fitted, the *R*_0_, *R*_s_ and *R*_ct_ are shown in Table 1.

**Table 1:** Fitted impedance value of the PAL and PVDF batteries

Impedance	*R*_0_ (Ω)	*R*_s_ (Ω)	*R*_ct_ (Ω)
PVDF-after the 1^st^ cycle	8.0	31.2	19.0
PAL-after the 1^st^ cycle	7.8	24.8	14.1
PVDF-after the 100^th^ cycle	11.6	51.6	52.8
PAL-after the 100^th^ cycle	8.5	25.5	23.6

**Fig. 4:**
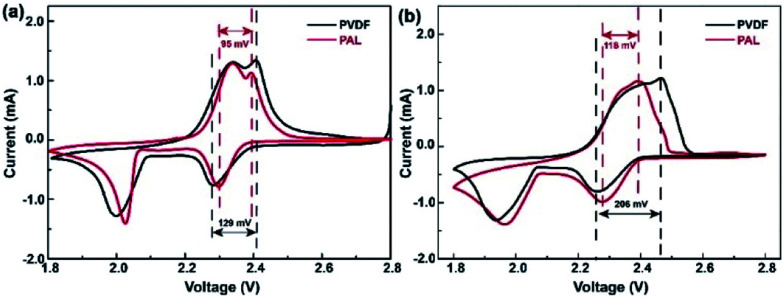
The *CV* curves of the PAL and PVDF batteries (a) in the 1^st^ cycle and (b) in the 100^th^ cycle at 0.2 mV s^−1^.

Both the PAL and PVDF batteries have almost the same *R*_0_ after the first cycle. The *R*_s_ and *R*_ct_ of PAL batteries are a little smaller than those of the PVDF battery and there is a difference of 6.4 and 4.9 Ω between these two kinds of batteries. The composition of the cathode material is the same except for the binder. Therefore, the difference in resistance should originate from the binders. PVDF is used as a solution dissolved in NMP with high viscosity (∼320 mPa s at 60 rpm). Compared with PVDF, a PAL binder is used as a water-based latex with low viscosity (∼58 mPa s at 60 rpm). The low viscosity means that it is helpful for a uniform dispersion of PAL in the cathode material, relation to the smaller *R*_s_ and *R*_ct_ in PAL battery. The difference in internal resistance can also be verified from the polarization in *CV* curves as shown in Fig. 4a. At the higher voltage peak, the polarization of the PAL battery between the reversible redox pairs is 95 mV, being much smaller than that of the PVDF battery (129 mV). The examined larger polarization in the PVDF battery should originate from the larger *R*_s_ and *R*_ct_. The *CV* at a variable scan rate show that the larger diffusion coefficient and faster Li^+^ transport present in the PAL cathode, in agreement with the examined smaller resistance and polarization (Fig. S7†).100 cycles later, the *R*_s_ and *R*_ct_ only increase by 0.7 and 9.5 Ω in the PAL battery, much less than those in the PVDF battery (20.4 and 33.8 Ω, respectively). The polarization in *CV* curves can also verify the increase in internal resistance (Fig. 4b). The polarization between the redox pairs at a high voltage peak is 118 mV in the PAL battery, less than that of the PVDF battery (206 mV). After 100 cycles, the polarization voltage increases 23 mV in the PAL battery, much less than that in the PVDF battery (77 mV), which means that the binder has affected the discharge behavior of the sulfur cathode.

To investigate the effect of the binder while discharging, the battery is directly discharged to 1.5 V in the first cycle, the potential of forming Li_2_S.^18,63,64^ As shown in Fig. S8,† another long discharge plateau at 1.76 V with capacities of about 210 mA h g^−1^ appears in the PAL battery corresponding to the conversion from Li_2_S_*n*_ to Li_2_S_2_, while only a shorter discharge plateau at 1.73 V in the PVDF battery is observed, implying the incomplete conversion of Li_2_S_*n*_.^65^ The discharge slop between 1.76 V and 1.55 V could be assigned to the electrolyte additives reduction and the solid phase transformation from Li_2_S_2_ to Li_2_S. Much larger discharge capacity is also obtained in the PAL battery, indicating the easy conversion from Li_2_S_2_ to Li_2_S with the PAL binder. XPS analysis reveals that S^2−^ species are the main discharge products in the PAL cathode, while the principle discharge products are S_2_^2−^ with a certain number of S^2−^ displayed in PVDF cathode (Fig. S9†).^66,67^ The results demonstrate an obvious advantage of PAL over PVDF, promoting the conversion of polysulfides.

**Fig. 5:**
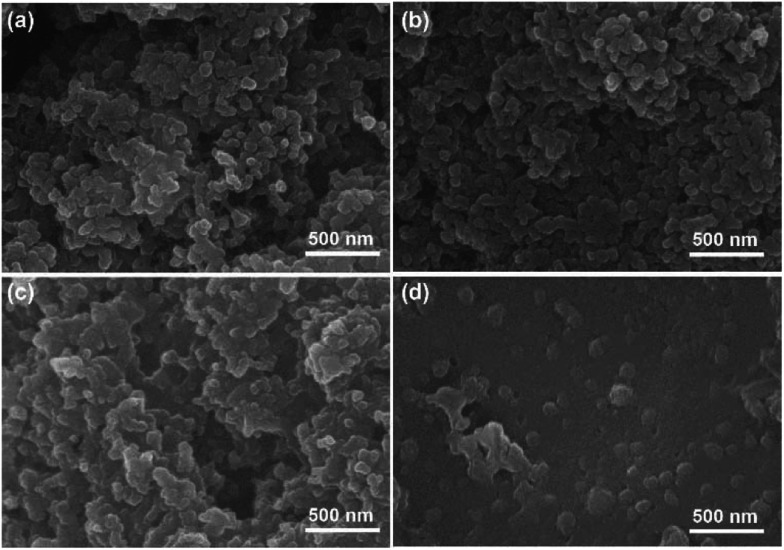
The surface morphologies of cathode for PAL battery after (a) the 1^st^ cycle and (c) the 100^th^ cycle. The surface morphologies of cathode of PVDF battery after (b) the 1^st^ cycle and (d) the 100^th^ cycle.

Morphologies of the PAL and PVDF cathodes are studied with SEM. As shown in Fig. 5a, b and S10a–d† (EIS), the fine particles of carbon/sulfur composite with granular aggregation are discovered in two types of cathodes after the 1^st^ cycle, and no obvious difference is found. After the 100^th^ cycle, the morphologies of PAL cathode have little change (Fig. 5c, S10e and f† (EIS)). The granular particles still exist except that some small particles fuse together to form larger ones due to the formation of insoluble discharge products. It's well known that moderate crosslinking can provide elasticity for rubber to recover deformation.^68^ Considering the huge volumetric expansion and shrinkage of sulfur cathode during cycling,^52,53^ the elasticity originated from the crosslinking PAL binder should have played a very important role in keeping the granular cathode structure stable. The PAL binder can endure periodic tensile stretching for many cycles up to the ratio of 200% (Fig. 1c), meaning excellent recovery to the expansion and shrinkage deformation of the sulfur cathode. So the granular cathode structure can still be observed after 100 cycles. However, the morphologies of PVDF cathode change greatly after 100 cycles (Fig. 5d, S10g and h† (EIS)). The granular aggregation morphology almost disappears and a dense layer covers the surface. The dense layer, resulting in large *R*_s_ increase, can be attributed to the heavy aggregation of insoluble discharge products such as Li_2_S_2_ and Li_2_S.^8,69^

**Fig. 6:**
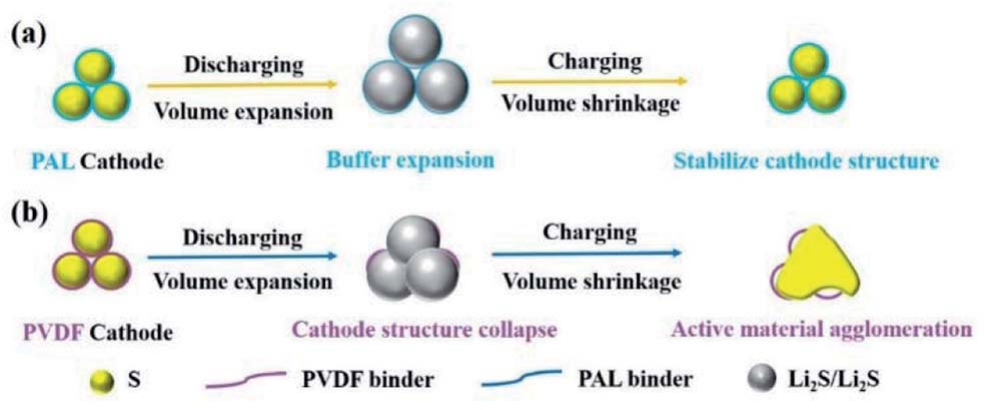
Schematic illustration of the structure and mechanism of PAL and PVDF binders in Li–S batteries.

The mechanism of the binder affecting the cathode structure is illustrated in Fig. 6. As the PAL binder with high resilience is used, the elasticity might help the binder adhere well to the sulfur particles no matter of the expansion or shrinkage that occurs, which can help stabilize the cathode granular structure (Fig. 6a). The stable granular structure in the cathode can facilitate Li^+^ transfer and diffusion in the cathode, leading to a low increase rate of both the *R*_s_ and *R*_ct_ (Fig. 3a and Table 1). When a plastic binder is used, the deformation of PVDF can't match well with the volumetric changes of sulfur particles in the cathode (Fig. 6b). The reduced polysulfides might migrate from the PVDF binder layer and fuse together. After several cycles, the granular structure gradually changes to dense aggregation in the cathode, resulting in the collapse of the cathode structure (Fig. 5d), large *R*_s_ and *R*_ct_ (Fig. 3b and Table 1) and inconvenient for Li^+^ transfer.

## Conclusions

A novel water-based PAL binder with self-crosslinking function groups is synthesized for sulfur cathode *via* emulsion polymerization. The as-prepared PAL possesses nano-sized particles, low *T*_g_, easily achieving a uniform dispersion and good adhesion in the cathode. Furthermore, the crosslinking PAL binder can provide fine elasticity to endure huge volumetric change and keep the granular cathode structure stable during cycling. The PAL battery shows excellent cycle and C-rate performance, less increase in internal resistance and lower polarization, which is the benefit of the stable cathode structure.

## Conflicts of interest

There are no conflicts of interest to declare.

## Supplementary Material

RA-009-C9RA08238G-s001
