# Supplementary material for: Stabilizing cathode structure via the binder material with high resilience for lithium–sulfur batteries
Source: RSC Adv. 2019 Dec 6;9(69):40471–7. doi: 10.1039/c9ra08238g (PMC9076401; doi:10.1039/c9ra08238g)
Supplement: RA-009-C9RA08238G-s001 [file RA-009-C9RA08238G-s001.pdf]

Electronic Supplementary Material (ESI) for RSC Advances.
This journal is © The Royal Society of Chemistry 2019

Supplementary Information

Stabilizing Cathode Structure *via* the Binder Material with High Resilience for Lithium-Sulfur Batteries

Fengquan Liu[†], *Zhiyu Hu*[†], *Jinxin Xue, Hong Huo, Jianjun Zhou*[*], *Lin Li*

Beijing Key Laboratory of Energy Conversion and Storage Materials, College of Chemistry, Beijing Normal University, Beijing, China 100875

[†]Fengquan Liu and Zhiyu Hu contribute equally for this study.

Correspondence and requests for materials should be addressed to J. J. Zhou.
(pla_zjj@bnu.edu.cn)

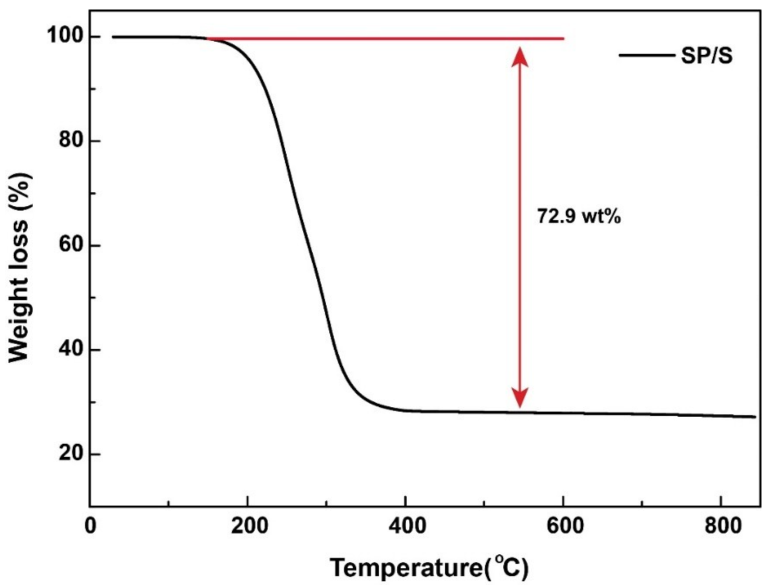

Fig. S1. The TGA curve of the SP/S composite cathode.

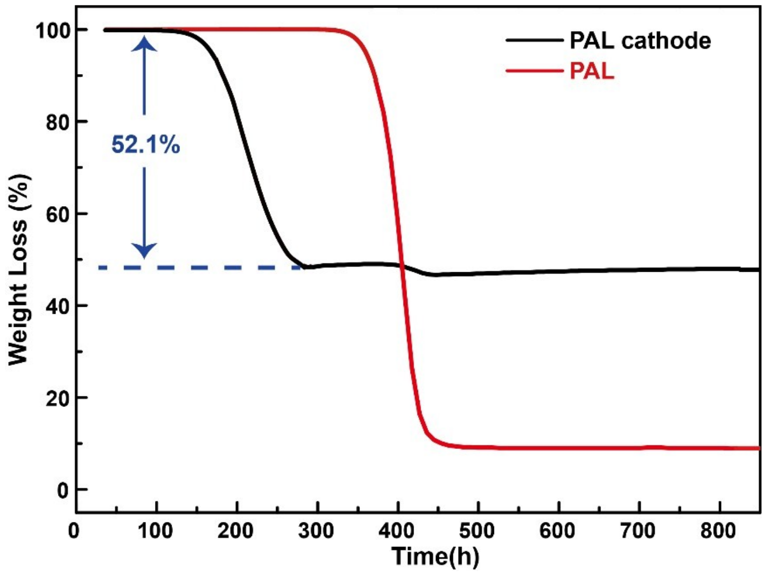

Fig. S2. The thermogravimetric curves of the PAL and PAL cathode after vacuum drying.

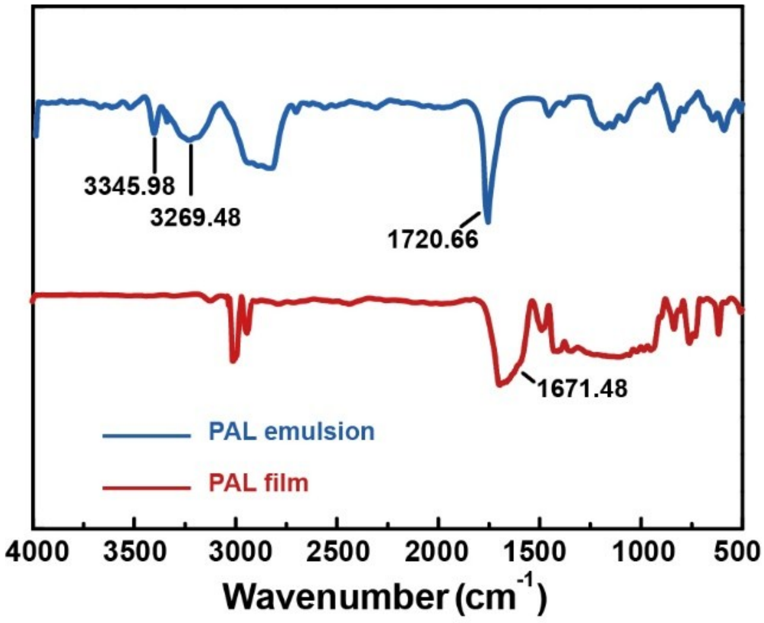

Fig. S3. The FTIR spectra of the PAL emulsion and PAL film.

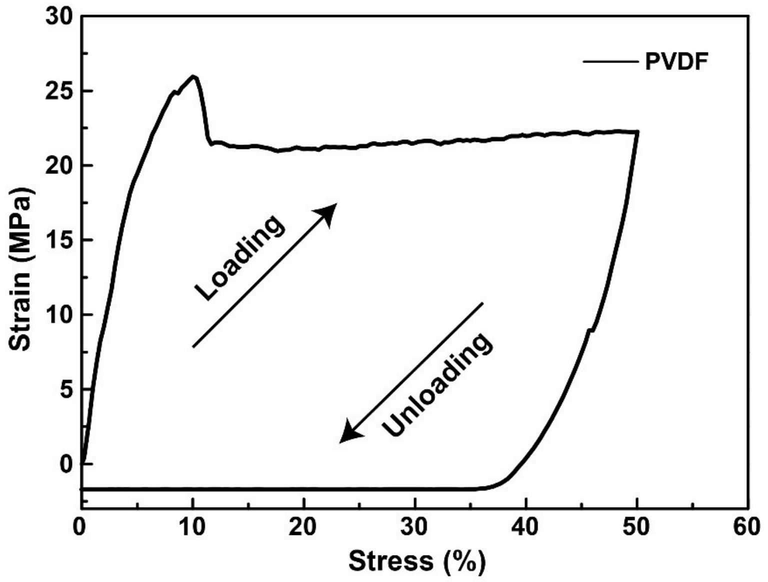

Fig. S4. Cyclic loading-unloading curves of PVDF film when the maximum tensile strain is 50%.

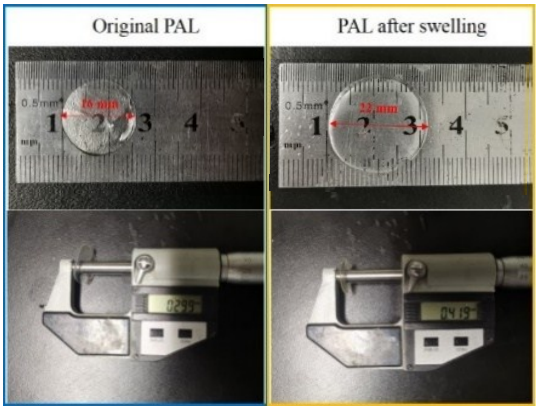

Fig. S5. Images of the PAL disk before and after swelling.

As shown in Fig. S5†, a disk of PAL membrane was prepared and immersed in the liquid electrolyte (1 M LiTFSI DOL/DME (v/v, 1/1) with 1 wt% $LiNO_3$). After 24 hours, the diameter of the disk increased from 16 mm to 22 mm, and the thickness increased from 0.299 mm to 0.419 mm, corresponding to about 40% increase in both the diameter and thickness.

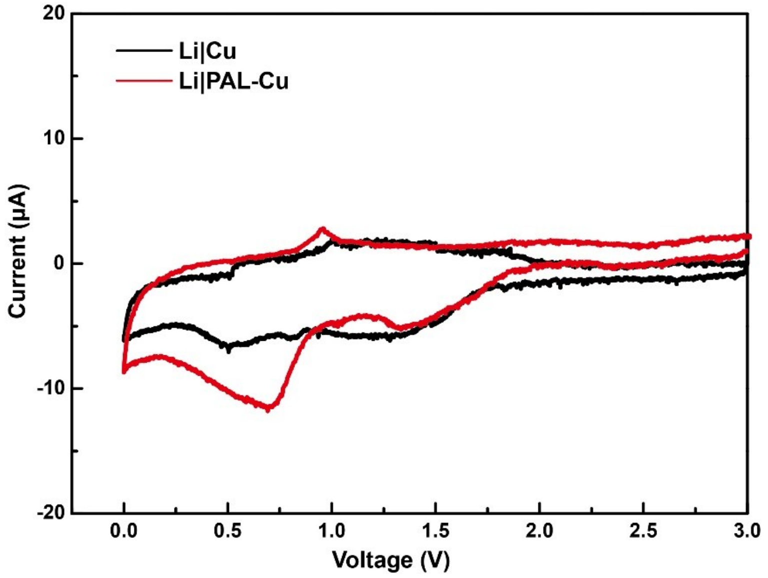

Fig. S6. The CV profiles of Li|Cu and Li|PAL-Cu batteries with a scan rate of 0.1 mV/s .

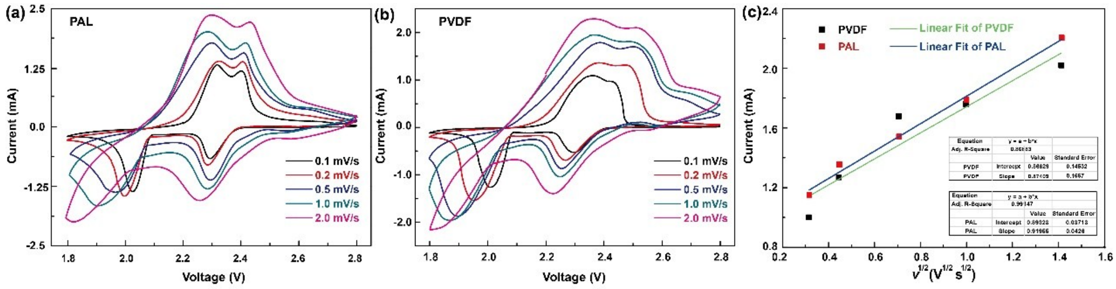

Fig. S7. The CV profiles of (a) the PAL and (b) PVDF battery at the varied scanning rates from 0.1 mV s^{-1} to 2.0 mV s^{-1}. (c) Linear fits of PAL and PVDF batteries between peak current and the square root of the scanning rate.

CV is scanned at variable rate to study the positive effect of the PAL binder (Figs. S7(a-b), ESI†). The apparent chemical diffusion coefficient of Li ion (D_{Li^+}) can be calculated by the Randles-Sevick equation.

$$I_p = 2.69 \times 10^5 n^{3/2} C_{Li^+} A D_{Li^+}^{1/2} v^{1/2}$$

Where I_p is the peak current, n is the charge transfer number (for Li-S battery, n=2), A is the surface area of electrode, C_{Li^+} is Li ion concentration in unit volume, v is the scan rate (V s^{-1}). The peak current can be linearly fitted with the square root of the scanning rate (Fig. S7(c), ESI†). The slope of fitting linear is 0.950 and 0.864 for the PAL and PVDF cathodes, respectively. Because other parameters such as n, C_{Li+} and A can be regarded as constants, D_{Li^+} is proportional to the slope, which means that the slope can be used as a measure of the D_{Li^+}. For PAL and PVDF cathodes, the D_{Li^+} ratio is 1.10, indicating larger diffusion coefficient and faster Li^+ transport in PAL cathode, verifying the role of stable cathode structure in promoting the diffusion and transfer of Li^+.

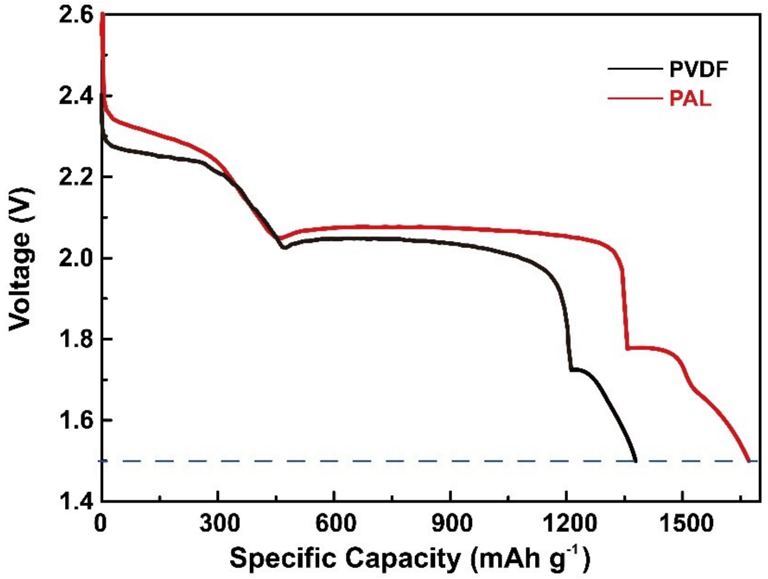

Fig. S8. The discharge profiles of different sulfur cathodes discharge to 1.5 V.

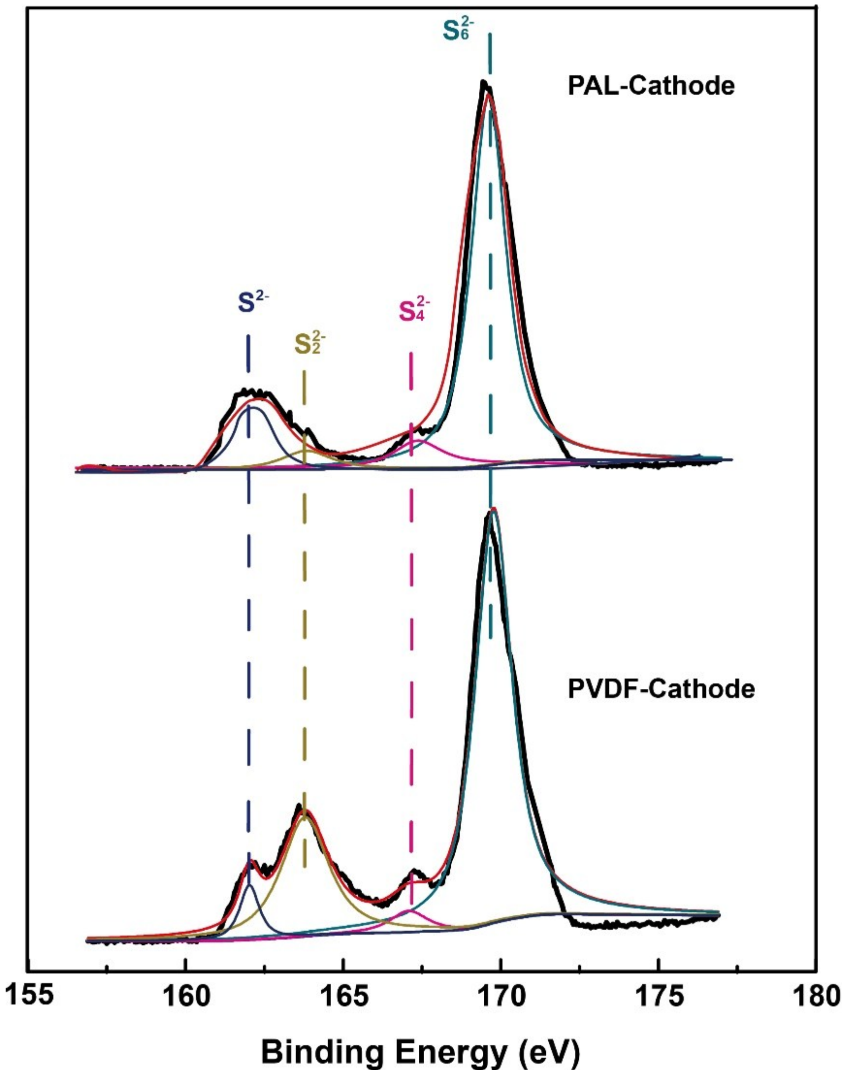

Fig. S9. The XPS spectra of S2p for different sulfur cathodes discharge to 1.5 V.

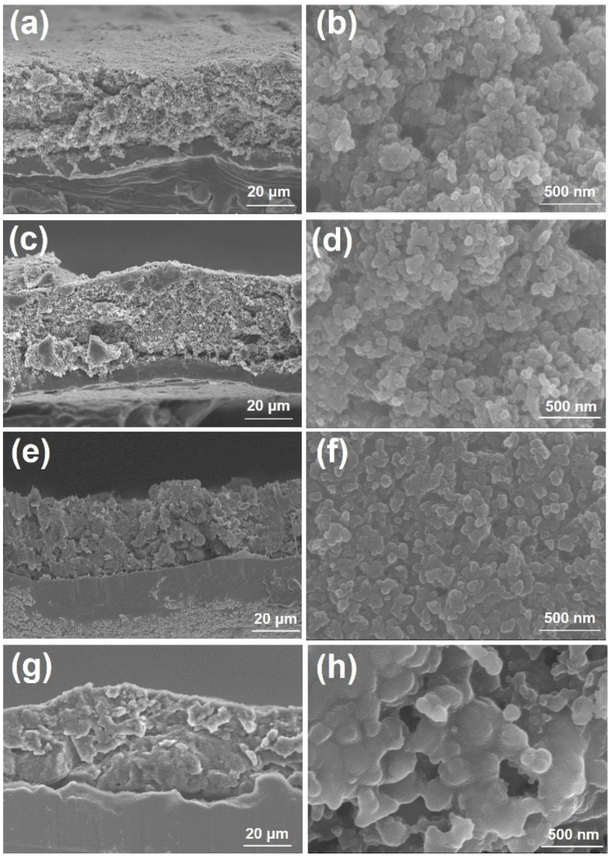

Fig. S10. The cross-sectional morphologies of (a,b) PAL and (c,d) PVDF cathodes after the 1st cycle. The cross-sectional morphologies of (e,f) PAL and (g,h) PVDF cathodes after the 100th cycle.

Table S1 The electrochemical performance of Li-S batteries with different binders.

Type of binder	Discharge capacity	Capacity retention ratio	Ref.
PPA	~763 mAh g^{-1}, 100 cycles at 0.2 C.	93%	40
PEG-PEO	~650 mAh g^{-1}, 50 cycles at 0.2 C.	54.2%	41
PVP	~714 mAh g^{-1}, 200 cycles at 0.2 C.	94%	42
Na-alginate	508 mAh g^{-1}, 50 cycles at 0.2 C.	64.5%	43
Gelatin	626 mAh g^{-1}（for FD-cathode）and 408 mAh g^{-1}（for ETD-cathode）, 50 cycles at 0.12 C.	50.7%（for FD-cathode）<br>36.0%（for ETD-cathode）	44
PEI	554 mAh g^{-1}, 300 cycles at 0.5 C.	82.7%	45
PAA	735 mAh g^{-1}, 100 cycles at 0.5 C.	49%	46
GO-PAA	635 mAh g^{-1}, 100 cycles at 0.5 C.	77.4%	47

TA/PEO	476.7 mAh g^{-1}, 1000 cycles at 0.2 C	67%	48
APP	640 mAh g^{-1}, 400 cycles at 0.5 C	87%	49
PTMA	625 mAh g^{-1}, 500 cycles at 100 mA g^{-1}.	75%	50
PAL	802 mAh g^{-1}, 100 cycles at 0.1 C.	62.1%	This paper
